# Evaluating the Association Between Current Smokers and Myocardial Infarction: A Cross-Sectional Study

**DOI:** 10.7759/cureus.90565

**Published:** 2025-08-20

**Authors:** Meenakshi R Yathindra, Grace O Odero, Sai Sudhamsh Govindu, Ritwik Sharma, Kazuaki Sato, Tejeet Reddy Mettu, Ajinkya Vijay Mahorkar

**Affiliations:** 1 Internal Medicine, Kasturba Medical College, Mangaluru, IND; 2 Anesthesiology, Coptic Hospital, Nairobi, KEN; 3 Internal Medicine, Gandhi Medical College, Secunderabad, IND; 4 Cardiovascular Surgery, GNRC Hospital, Guwahati, IND; 5 Emergency Medicine, Yokota Air Base Hospital (374th Medical Group), Fussa, JPN; 6 Internal Medicine, Andhra Medical College, Visakhapatnam, IND; 7 Cardiology, Avanti Institute of Cardiology, Nagpur, IND

**Keywords:** brfss, cross-sectional study, myocardial infarction (mi), odds ratio, retrospective study, smoking

## Abstract

Background

Smoking contributes to myocardial infarction (MI) by causing endothelial damage, accelerating atherosclerosis, and increasing the risk of thrombosis. Given the high prevalence of smoking in the population, assessing its association with MI is essential. Hence, this study aimed to evaluate the association between active smoking and MI by assessing its prevalence in smokers versus non-smokers, based on demographic and socioeconomic characteristics.

Methodology

This retrospective, cross-sectional study utilized the 2022 Behavioral Risk Factor Surveillance System database. The disease variable was MI, and the risk factor was smoking. Control variables included demographic characteristics (age, gender, and race) and socioeconomic factors (education and income). Data were analyzed using cross-tabulation, with results expressed as odds ratios (ORs) and confidence intervals (CIs).

Results

The total number of participants involved was 407,126. Among them, 49,504 were reported as smokers, and 357,622 were reported as non-smokers. Participants who reported being current smokers had reported higher odds of reporting having MI compared to non-smokers (8.4% vs. 5.3%, OR = 1.627, CI = 1.571-1.685). This was observed across all age groups, with the highest risk observed in the smokers in the 18-24-year age group (OR = 6.15; 95% CI = 3.926-9.434), and the risk was inversely proportional to age. Gender analysis revealed that the odds of MI in current female smokers were 1.823 (95% CI = 1.724-1.927) compared to 1.464 (95% CI = 1.399-1.532) in their male counterparts. Racial stratification revealed that the smokers who belonged to the non-Hispanic/other racial group had a relatively higher OR of 2.002 (95% CI = 1.826-2.191) when compared to the Black non-Hispanic (OR = 1.737, 95% CI = 1.525-1.976) and white non-Hispanic (OR = 1.579; 95% CI = 1.516-1.645) groups. Regarding socioeconomic factors, smokers with advanced education were more likely to report MI compared to those with basic education, with ORs of 1.685 (95% CI = 1.603-1.771) and 1.324 (95% CI = 1.259-1.393), respectively. Participants earning over $50,000 annually had higher odds of MI among smokers (OR = 1.442; 95% CI = 1.339-1.553) than those earning less than $50,000 annually (OR = 1.333; 95% CI = 1.273-1.396).

Conclusions

The findings of the study revealed that smoking had a strong association with MI across demographic and socioeconomic groups.

## Introduction

Myocardial infarction (MI) occurs when there is reduced blood flow through the coronary arteries, causing damage to the myocardium from the resulting ischemia. The reduced oxygen supply injures cardiac muscle tissue, which results in a heart attack [1]. In 2019, cardiovascular disease (CVD) resulted in 18.6 million deaths worldwide, which represented 49.2% of total deaths, making CVD the leading cause of death globally. The high prevalence of MI, together with its high fatality rates and unfavorable outcomes, worsens because of the growing number of older people in the population [2]. According to the World Health Organization (WHO), CVD impacts individuals from all socioeconomic backgrounds, geographic locations, and genders. Multiple factors, including cardiometabolic, behavioral, environmental, and social risk factors, contribute to the development of CVD. The development and progression of CVD strongly relate to three key modifiable risk factors, which include unhealthy diet, tobacco smoking, and physical inactivity [3].

Research has shown that cigarette smoking is an extremely preventable risk factor among all other risk factors. Multiple epidemiological studies demonstrate that smoking actively and being exposed to second-hand smoke both lead to higher rates of CVD and death [4]. The pathogenesis of CVD development among smokers is believed to be due to endothelial dysfunction, elevated myocardial oxygen demand, and a heightened risk of thrombosis [5]. The relationship between smoking and CVD remains well documented, but scientists have not fully identified the exact components of cigarette smoke and their biological mechanisms.

The Behavioral Risk Factor Surveillance System (BRFSS) is a yearly national telephone survey used in the United States. The BRFSS relies on self-reported information from U.S. residents about their preventive healthcare practices, chronic diseases, and health-related behaviors. With one of the largest population-based surveillance systems, the BRFSS is very useful in tracking public health trends and identifying potential associations between lifestyle factors and disease. This study aimed to explore the correlation between smoking and MI using the 2022 BRFSS database, analyzing how smoking influences MI incidents in various socioeconomic and demographic populations.

This study aimed to evaluate the odds of MI in the exposed group (current smokers) compared to the unexposed group (non-smokers), based on participants’ demographic (age, gender, race) and socioeconomic characteristics (annual income and education level).

## Materials and methods

This retrospective original research study was conducted using the BRFSS database [6]. Data for this study were extracted on April 1, 2025, and analyzed. As the BRFSS dataset contains de-identified public data, this research qualified as non-human participant research [7]. As a result, ethics committee approval was not required.

Data were extracted using the BRFSS Web-Enabled Analysis Tool (WEAT) for the year 2022. The primary disease variable in this study was MI, “Ever told you had a heart attack, also called a MI (CVDINFR4),” and the lifestyle risk factor variable was smoking, “Calculated variable for adults who are current smokers (_RFSMOK3).” Control variables included demographic and socioeconomic characteristics. The following demographic characteristics were included: age: calculated variable for four-level imputed age category (_AGE_G, 18-24, 25-44, 45-64, 65+), categorized into four groups (18-24, 25-44, 45-64, 65+); gender: (respondent’s sex), categorized as male and female; and race: calculated variable for four-level race groups, categorized as White (non-Hispanic), Black (non-Hispanic), and Hispanic/Other races. Socioeconomic characteristics included education level (EDUCA), categorized as basic education (high school or less) and advanced education (some college or higher), and annual income (_INCOMG1), categorized as <$50,000 and >$50,000. Participants who responded to the selected disease, risk factor, and control variables were included in the study, ensuring completeness of data for analysis.

Descriptive statistics, including numbers and percentages, were generated for each variable using cross-tabulation in the BRFSS WEAT. The data were exported to Microsoft Excel (Microsoft Corp., Redmond, WA, USA) for organization and further processing. Statistical analyses were performed using R version 4.3.1 (2023). Results were expressed as odds ratios (ORs) with 95% confidence intervals (CIs). A p-value <0.05 was considered statistically significant.

## Results

Of the total participants, current smokers had significantly higher odds of reporting a history of MI compared to non-smokers (8.4% vs. 5.3%, OR = 1.627; 95% CI = 1.571-1.685; p < 0.05), as shown in Table 1.

**Table 1:** Prevalence of current smokers in participants with self-reported MI. *: Significant. CI = confidence interval; OR = odds ratio; MI = myocardial infarction

Current smokers	MI			OR (CI)
	Yes	Yes	No	1.627 (1.571, 1.685)*
		4,156 (8.4%)	45,348 (91.6%)	
	No	19,068 (5.3%)	338,554 (94.7%)	

Age-specific analyses revealed a clear trend. The younger the age group, the higher the relative risk of MI among smokers. Participants aged 18 to 24 years had the highest odds, with current smokers in this group being over six times more likely to report MI compared to their non-smoking peers (OR = 6.15; 95% CI = 3.926-9.434). This elevated risk declined with age but remained significant across all groups. Among those aged 25-44 years, the OR was 3.151 (95% CI = 2.755-3.6); for those aged 45-64 years, it was 2.38 (95% CI = 2.25-2.517); and in participants aged 65 years and older, the OR was 1.624 (95% CI = 1.54-1.711). Gender-based analysis showed that female smokers had a higher relative risk of MI than male smokers. The odds of MI in current female smokers were 1.823 (95% CI = 1.724-1.927) compared to 1.464 (95% CI = 1.399-1.532) in their male counterparts. When stratified by race, the association between smoking and MI remained statistically significant across all groups. The highest risk was observed in participants identifying as non-Hispanic or belonging to other racial groups, with an OR of 2.002 (95% CI = 1.826-2.191). Black non-Hispanic smokers had an OR of 1.737 (95% CI = 1.525-1.976), while White non-Hispanic smokers showed a slightly lower but still significant risk (OR = 1.579; 95% CI = 1.516-1.645), as represented in Table 2.

**Table 2:** Prevalence of current smokers in participants with self-reported MI based on demographic characteristics. *: Significant. CI = confidence interval; OR = odds ratio; MI = myocardial infarction

	Current smokers	N	Participants with MI	Participants without MI	OR (CI)
Age groups					
18–24 years	Yes	1,594	32 (2%)	1,562 (98%)	6.15 (3.926, 9.434)*
	No	23,194	77 (0.3%)	23,117 (99.7%)	
25–44 years	Yes	14,549	349 (2.4%)	14,200 (97.6%)	3.151 (2.755, 3.6)*
	No	83,347	645 (0.8%)	82,702 (99.2%)	
45–64 years	Yes	21,239	1,903 (9%)	19,336 (91%)	2.38 (2.25, 2.517)*
	No	114,654	4,553 (4%)	110,101 (96%)	
65+ years	Yes	12,122	1,872 (15.4%)	10,250 (84.6%)	1.624 (1.54, 1.711)*
	No	136,427	13,793 (10.1%)	122,634 (89.9%)	
Gender					
Male	Yes	25,037	2,512 (10%)	22,525 (90%)	1.464 (1.399, 1.532)*
	No	167,110	11,827 (7.1%)	155,283 (92.9%)	
Female	Yes	24,467	1,644 (6.7%)	22,823 (93.3%)	1.823 (1.724, 1.927)*
	No	190,512	7,241 (3.8%)	183,271 (96.2%)	
Race					
White, non-Hispanic	Yes	35,022	3,058 (8.7%)	31,964 (91.3%)	1.579 (1.516, 1.645)*
	No	261,596	14,943 (5.7%)	246,653 (94.3%)	
Black, non-Hispanic	Yes	4,368	323 (7.4%)	4,045 (92.6%)	1.737 (1.525, 1.976)*
	No	26,810	1,178 (4.4%)	25,632 (95.6%)	
Non-Hispanic/Other race	Yes	8,573	651 (7.6%)	7,922 (92.4%)	2.002 (1.826, 2.191)*
	No	59,207	2,335 (3.9%)	56,872 (96.1%)	

Socioeconomic status also influenced the relationship between smoking and MI. Smokers with advanced education were more likely to report MI compared to those with basic education, with OR of 1.685 (95% CI = 1.603-1.771) and 1.324 (95% CI = 1.259-1.393), respectively. Income showed a similar pattern. Participants earning over $50,000 annually had higher odds of MI among smokers (OR = 1.442; 95% CI = 1.339-1.553) than those earning less than $50,000 (OR = 1.333; 95% CI = 1.273-1.396), as noted in Table 3.

**Table 3:** Prevalence of current smokers in participants with self-reported MI based on socioeconomic characteristics. *: Significant. CI = confidence interval; OR = odds ratio; MI = myocardial infarction

Socioeconomic characteristics	Current smokers	N	Participants with MI	Participants without MI	OR (CI)
Education level					
Basic education	Yes	23,606	2,197 (9.3%)	21,409 (90.7%)	1.324 (1.259, 1.393*
	No	97,564	7,016 (7.2%)	90,548 (92.8%)	
Advanced education	Yes	25,746	1,946 (7.6%)	23,800 (92.4%)	1.685 (1.603, 1.771)*
	No	258,662	11,969 (4.6%)	246,693 (95.4%)	
Annual income					
Less than $50,000	Yes	25,696	2,649 (10.3%)	23,047 (89.7%)	1.333 (1.273, 1.396)*
	No	111,539	8,853 (7.9%)	102,686 (92.1%)	
Greater than $50,000	Yes	16,327	847 (5.2%)	15,480 (94.8%)	1.442 (1.339, 1.553)*
	No	179,604	6,564 (3.7%)	173,040 (96.3%)	

## Discussion

This retrospective observational study evaluated the relationship between current smoking, a modifiable risk factor, and self-reported MI using the 2022 BRFSS database. Using responses from 407,126 adults nationwide, the study adjusted for demographic factors such as age, gender, and race, as well as socioeconomic factors such as income and education. The study discovered a statistically significant link between MI and current smoking, with current smokers at a higher risk of MI than non-smokers. Stratified analysis confirmed that this correlation applied to all age groups, sexes, racial categories, income levels, and educational backgrounds. The BRFSS, the world’s largest ongoing telephone health survey system, is conducted annually by the Centers for Disease Control and Prevention (CDC) and state health departments to collect state-level data on health risk behaviors, chronic conditions, and preventive health practices among adult Americans. BRFSS has combined landline and cell phone sampling since 2011. It generates representative population estimates that direct public health policies and activities by means of advanced weighting approaches [7].

Smoking is an acknowledged risk factor for CVD, particularly MI. The pathophysiological mechanisms include endothelial dysfunction, reduced oxygen transport from carbon monoxide exposure, increased thrombogenicity, and the promotion of atherogenesis [8,9]. These processes shape both acute and chronic coronary syndromes. Smoking is well known to cause atherosclerosis more rapidly and aggressively than non-smoking [9,10]. Moreover, nicotine raises blood pressure and heart rate by inducing catecholamine release, thus aggravating cardiovascular strain [10]. With 18.7% (46 million) of adults using tobacco products, including 11.5% who smoke cigarettes and 4.5% who use e-cigarettes, tobacco use remains a major preventable cause of disease and death in the United States. This data emphasizes the need for continuous, all-encompassing tobacco control programs to minimize tobacco-related damage and inequalities [11].

Relationship between smoking and cardiovascular mortality and myocardial infarction

With an OR of 1.627 (95% CI = 1.571-1.685), current smokers were far more likely than non-smoking individuals to report MI. As shown in Table 1, 8.4% of current smokers claimed to have a history of MI, compared to 5.3% of non-smoking individuals. These findings are consistent with earlier population-based studies, such as the INTERHEART study, which found that smoking increases more than one-third of MI risk worldwide [12,13]. Yusuf et al. (2004) found that current smokers had a population attributable risk of 35.7% and almost threefold higher risk of acute MI (OR = 2.87). For ex-smoking individuals, the risk dropped over time; nevertheless, a residual excess risk persisted even 20 years after quitting, and risk increased with increasing number of cigarettes smoked [12]. Teo et al. (2006) reported that smoking beedies and chewing tobacco greatly increase the risk of acute MI, with combined use carrying the highest risk (OR = 4.09). In a dose-dependent sense, secondhand smoke also increased the risk of acute MI (OR = 1.62) for exposure of more than 21 hours per week [13].

The Atherosclerosis Risk in Communities Study found a 40% reduction in risk for all-cause mortality (AHR = 0.60), while the Healthy Ageing Study found that quitting smoking was associated with a 65% reduction in risk (AHR = 0.35), supporting the cardiovascular mortality link. The Nurses’ Health Study found smokers to have an 8.17-fold higher risk of dying from CVD than non-smoking individuals [14]. Bucholz et al. noted that 14.8% of over 158,000 older Medicare patients with acute MI were current smokers, which resulted in a slightly lower short-term mortality (30-day HR = 0.91) but a significantly higher long-term mortality (17-year HR = 1.19). The long-term negative effects of smoking, even after an acute MI event, were supported by findings that smoking was linked to a lower life expectancy and significant years of life lost at all ages [15].

Variations in the risk of myocardial infarction linked with age, gender, and race/ethnicity: smoking

Table 2 shows the relationship between smoking and MI in different demographic groups. MI and smoking were strongly linked; the 18-24-year age group had the highest MI (OR = 6.15). This suggests that younger smokers may show more or earlier manifestations of cardiovascular damage. The OR fell with age but stayed significant across all age groups; it was the highest in the 25-44-year age group and the lowest in the 65+-year age group (OR = 1.624). Alzahrani et al.’s analysis of data from the 2014 and 2016 National Health Interview Surveys showed daily e-cigarette use was independently linked to higher odds of myocardial infarction (OR = 1.79; 95% CI = 1.20-2.66; p = 0.004), even after controlling for traditional cigarette smoking and other cardiovascular risk factors. Daily traditional cigarette smoking (OR = 2.72; 95% CI = 2.29-3.24; p = 0.001) carried an even higher risk. Further risk factors were hypertension, high cholesterol, diabetes, and advancing age; women were less likely to be at risk (OR = 0.47; p = 0.001) [16].

Males showed a significantly lower OR for MI (OR = 1.464) compared to females (OR = 1.823), indicating that smoking had a stronger association with MI in women. With an excess risk two to four times higher than that of men, smoking increases women’s risk of coronary heart disease (CHD), showing a dose-dependent relationship [17]. Three to five years of abstinence causes this risk to start to drop and resemble that of non-smokers. While in diabetic men the risk is raised by two to three times, smoking increases the risk of coronary death in diabetic women by three to seven times. This is because smoking compromises the protective qualities of estrogen. Smoking also contributes to the stronger association between hypertension, CHD, and early mortality seen in women [17]. A study by Kim et al. on 12,565 Korean acute MI patients who were current smokers found that women had notably higher rates of early (30-day) and two-year all-cause death as well as recurrent MI even after drug-eluting stent implantation [18]. A sizable prospective cohort study including more than 24,000 participants in Copenhagen found that female smokers had a notably higher relative risk of MI (2.24) than male smokers (1.43) [19]. This variation indicated that women are more biologically sensitive regardless of age group or degree of tobacco use [19]. Weiner et al. reported that younger smokers (31-50 years) had a higher mortality rate than non-smoking individuals in the same group, and current smokers had their first acute MI more than 10 years earlier than non-smoking individuals (56.6 years vs. 70.4 years for men and 66.8 vs. 70.4 years for women) [20].

Every racial group revealed a noteworthy relationship between smoking and MI. The Non-Hispanic/Other race group had the highest OR (2.002), followed by Black, non-Hispanic (OR = 1.737) and White, non-Hispanic (OR = 1.579). The National Health and Nutrition Examination Survey 1999-2018 data showed that 21.7% of respondents currently smoked; rates were higher among Black people (25.5%), younger adults, and men than among White people (22.0%), Hispanic people (18.6%), and other groups (19.1%). Particularly, Black and Hispanic younger people with lower average ages were more likely to smoke. Underprivileged subgroups had higher smoking prevalence; men had more than women. Specifically, 30.9% of Black participants with ≥5 unfavorable social factors who smoked, compared to 10.0% of similarly disadvantaged White participants, 67.8% of low-income Black participants, and 75.6% of Hispanic participants who smoked. These patterns show the need for concentrated, equity-oriented public health projects [21].

Although smoking is more common among white adolescents (18.6%) than African American adolescents (8.2%), the differences linger into adulthood. African Americans are more likely to be exposed to secondhand smoke and have lower rates of quitting, mostly due to the predominance of more addictive mentholated cigarettes. Tobacco companies have aggressively targeted menthol products to African Americans, as 71% of African American smokers use mentholated cigarettes compared to 21% of white smokers. This inclination drives African Americans to have reduced rates of quitting [22].

Variations in the risk of myocardial infarction linked with smoking based on the socioeconomic level

Table 3 demonstrates that people with lower socioeconomic status, particularly those who have had MI, smoke more frequently. Smoking rates were 9.3% among patients with MI and 7.2% among those without MI who had only a basic education (high school or less) (OR = 1.324). Those with advanced education, that is, more than college-level education, had lower rates (7.6% vs. 4.6%, OR = 1.685). Similarly, smoking rates were higher among those earning less than $50,000 per year (10.3% with MI vs. 7.9% without MI, OR = 1.333) than among those earning more (5.2% vs. 3.7%, OR = 1.424). As shown in Table 3, people with MI and lower socioeconomic status smoked more. Lower socioeconomic status was strongly associated with smoking; smoking rates were significantly higher in the unemployed, low-income, low-education, and uninsured individuals [23].

Lower socioeconomic status adults, especially those with Medicaid or no insurance, were more likely to smoke, according to Cornelius et al. (2021) [24]. Individuals diagnosed with depression also smoked more; this subgroup clearly showed racial and ethnic disparities. The study claimed that tobacco advertising disproportionately targeted low-income neighborhoods and communities with a high proportion of Black or American Indian/Alaska Native people, which can help explain the higher smoking prevalence [24].

According to the American Heart Association, low income magnifies the negative impact of modifiable risk factors, including smoking, on cardiovascular health. Still, CVD, one of the main causes of death in the United States, has clear variations linked to social determinants of health, particularly socioeconomic status. Lower socioeconomic status, that is, occupation, income, and education, is consistently linked to higher CVD risk, incidence, and death. Those with basic education have 1.3-1.7 times the risk of CVD when compared to individuals with advanced education. Lower-income earners are also more likely to smoke, eat poorly, and have less access to healthcare, all of which raise their risk of CVD. Racial and ethnic minorities, especially Black and Hispanic populations, face more burdens from CVD because of higher rates of hypertension, mortality, care barriers, and less supportive surroundings. Reducing inequalities and enhancing cardiovascular outcomes call for addressing structural elements, including poverty, discrimination, and lack of access to care [11].

Mead et al. found that between the ages of 18 and 44 years, Florida’s smoke-free laws reduced the prevalence of acute MI in non-Hispanic white adults. This result emphasizes the need for more investigation into secondhand smoke exposure in minorities and stronger policies to resolve racial inequalities in acute MI. Heart disease ranks as the most common cause of death in the United States, and acute MI is more likely in African American men and women than in non-Hispanic whites. Though smoking rates are lower, African Americans are more likely to be exposed to secondhand smoke; thus, AMI and CHD remain a major risk factor for this population [25].

The most significant result was that current smokers had 1.627 times the odds of having a MI when compared to non-smoking individuals, supporting the strong link between tobacco use and heart disease. The strength of this relationship changed significantly depending on socioeconomic and demographic elements [22].

In 2010, Stringhini et al. looked at how smoking and other health behaviors help explain the social inequality in mortality. Their analysis revealed that smoking accounted for 42% of the baseline gradient in all-cause mortality, 29% of the gradient in CVD, and 61% of the gradient in non-cancer/non-CVD causes, contributing significantly to mortality disparities. Though smoking rates dropped gradually, their effect on mortality inequality persisted, highlighting the long-term effects, especially for diseases such as lung cancer and chronic obstructive pulmonary disease, which can take decades to show symptoms [26].

Sharma et al. systematically reviewed and meta-analyzed four studies comprising 585,306 participants to assess the link between e-cigarette use and MI. E-cigarette users had higher odds of MI than non-users (OR = 1.33; 95% CI = 1.14-1.56), even if their odds were lower than those of conventional smokers (OR = 0.61; 95% CI = 0.40-0.93). This suggests that even if e-cigarettes may be less harmful, they still carry cardiovascular hazards [27].

Limitations

This study has several limitations. Report mistakes and recall bias could have been introduced, as the BRFSS is a self-reported telephone survey. The data ignores the degree and timing of the MI as well as clinical confirmation of the MI diagnosis. Moreover, the underrepresentation of individuals without landlines or who lacked fluency in Spanish or English could restrict the generalizability of the findings. A prospective cohort design would be more suitable to evaluate the causal links between smoking and MI.

## Conclusions

This study highlighted a higher likelihood of MI in current smokers across various demographic and socioeconomic groups. The likelihood was notably increased among young adults (especially those aged 18-24 years), female participants, and individuals of non-Hispanic/other racial backgrounds. Socioeconomic characteristics also revealed disparities, with elevated risk observed even among smokers with advanced education and higher income levels. Future research should focus on prospective studies to evaluate how smoking modifies the course of MI. Such studies would help formulate guidelines on smoking cessation and risk stratification, considering physical, behavioral, and sociodemographic factors.
